# SMYD3 promotes hepatocellular carcinoma progression by methylating S1PR1 promoters

**DOI:** 10.1038/s41419-021-04009-8

**Published:** 2021-07-23

**Authors:** Heyun Zhang, Zhangyu Zheng, Rongqin Zhang, Yongcong Yan, Yaorong Peng, Hua Ye, Lehang Lin, Junyao Xu, Wenbin Li, Pinbo Huang

**Affiliations:** 1 Department of Hepatobiliary Surgery, Sun Yat-sen Memorial Hospital, Sun Yat-sen University, 510120 Guangzhou, China; 2 Guangdong Provincial Key Laboratory of Malignant Tumor Epigenetics and Gene Regulation, Medical Research Center, Sun Yat-sen Memorial Hospital, Sun Yat-sen University, 510120 Guangzhou, China; 3 Department of Nuclear Medicine, The Sixth Affiliated Hospital, Sun Yat-sen University, 510655 Guangzhou, China; 4 Department of Nuclear Medicine, Sun Yat-sen University Cancer Center, State Key Laboratory of Oncology in South China, Collaborative Innovation Center for Cancer Medicine, Guangzhou, China; 5 Department of Pancreaticobiliary Surgery, Sun Yat-sen Memorial Hospital, Sun Yat-sen University, 510120 Guangzhou, China; 6 Department of Preventive Health Care, Sun Yat-sen Memorial Hospital, Sun Yat-sen University, 510120 Guangzhou, China

**Keywords:** Liver cancer, Oncogenes

## Abstract

Hepatocellular carcinoma (HCC) is one of the most common malignancies worldwide. SET and MYND domain-containing protein 3 (SMYD3) has been shown to promote the progression of various types of human cancers, including liver cancer; however, the detailed molecular mechanism is still largely unknown. Here, we report that SMYD3 expression in HCC is an independent prognostic factor for survival and promotes the proliferation and migration of HCC cells. We observed that SMYD3 upregulated sphingosine-1-phosphate receptor 1 (S1PR1) promoter activity by methylating histone 3 (H3K4me3). S1PR1 was expressed at high levels in HCC samples, and high S1PR1 expression was associated with shorter survival. S1PR1 expression was also positively correlated with SMYD3 expression in HCC samples. We confirmed that SMYD3 promotes HCC cell growth and migration in vitro and in vivo by upregulating S1PR1 expression. Further investigations revealed that SMYD3 affects critical signaling pathways associated with the progression of HCC through S1PR1. These findings strongly suggest that SMYD3 has a crucial function in HCC progression that is partially mediated by histone methylation at the downstream gene S1PR1, which affects key signaling pathways associated with carcinogenesis and the progression of HCC.

## Introduction

Hepatocellular carcinoma (HCC) comprises 75–85% of all primary liver cancers and is estimated to be one of the most common malignancies worldwide. Although the incidence and mortality have been decreasing in Asia, liver cancer is still the third-highest cause of cancer-related death [1]. With an increasing number of studies focusing on molecular mechanisms, more pathways and signaling factors related to carcinogenesis and metastasis have been discovered [2–5]. However, many problems related to genetic and epigenetic alterations in hepatocarcinogenesis remain unknown, and further research is required to better understand this issue.

SET and MYND domain-containing protein 3 (SMYD3), a member of the SET and MYND domain family of lysine methyltransferases, is overexpressed in various types of human cancers, including prostate cancer, breast cancer, colon cancer, colorectal cancer, ovarian cancer, and HCC [6–10]. Within SMYD3, the SET domain meditates the histone methylation of lysine residues, while the MYND domain contains a zinc finger motif that binds proline-rich regions and facilitates protein–protein interactions [11, 12]. Overexpression of SMYD3 plays an important role in the proliferation, adhesion, invasion, and migration of cancer cells, whereas decreases in SMYD3 expression inhibit cell growth, migration, invasion, and apoptosis [13–15]. Recently, methylation of MAP3K2 at lysine 260 by SMYD3 was shown to block the interaction between the PP2A phosphatase complex and MAP3K2, resulting in the activation of the MAP kinase signaling cascade and subsequent promotion of Ras-driven tumorigenesis [16]. In addition, SMYD3-mediated lysine 14 methylation in the PH domain of AKT1 enhances the phosphorylation of threonine 308 in AKT1, which promotes growth [17]. Furthermore, some studies have revealed that SMYD3 catalyzes histone H4 lysine 5 methylation to maintain transformed cell phenotypes and promotes cell cycle progression and cancer proliferation by dimethylating histone H2A.Z.1 at lysine 101 [18, 19]. The most frequent histone methylation event mediated by SMYD3 is on lysine 4 of H3; this modification is related to carcinogenic processes, such as proliferation, adhesion, invasion, and migration. Based on the results of these studies, SMYD3 might influence oncogenesis by altering the transcription of downstream target genes via methylation, especially histone methylation.

According to several studies, SMYD3 deregulation is common in HCC and is associated with aggressive biological characteristics, such as increased cell proliferation and migration and reduced apoptosis [13, 20]. However, the specific mechanisms contributing to the carcinogenesis and progression of HCC remain unclear, and the target genes of SMYD3 that are essential for transcriptional deregulation must be further characterized. In addition, correlations between SMYD3 expression and clinical features in patients with HCC have not been fully described.

Therefore, our study aimed to investigate the correlations between SMYD3 expression and both pathological features and clinical characteristics of patients with HCC and to search for potential downstream target genes of SMYD3 to elucidate the possible mechanism by which SMYD3 drives hepatocarcinogenesis.

## Results

### SMYD3 was overexpressed and associated with aggressive behaviors in HCC tissues

**Fig. 1:**
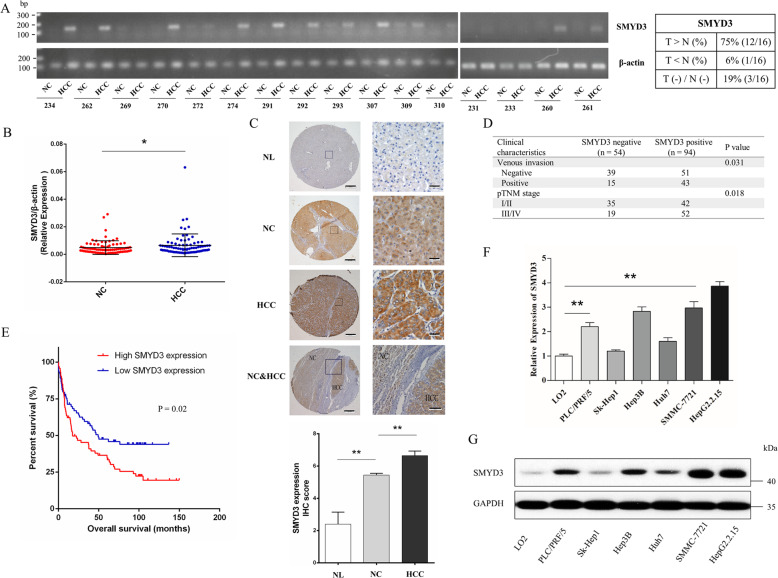
SMYD3 was overexpressed and associated with aggressive behaviors in hepatocellular carcinoma (HCC) tissues. **A** Reverse transcription-polymerase chain reaction (RT-PCR) analysis of SMYD3 mRNA expression levels in paired HCC and adjacent noncancerous tissues from 16 patients. **B** Quantitative real-time polymerase chain reaction (qRT-PCR) analysis of SMYD3 mRNA expression levels in 80 paired HCC and adjacent noncancerous tissue samples. **C** Immunohistochemistry (IHC) analysis of SMYD3 expression in 10 normal liver samples and 148 pairs of HCC and adjacent noncancerous tissue samples. **D** Correlations between SMYD3 expression and the clinicopathological characteristics of patients with HCC were determined using the chi-square test. SMYD3-negative was defined as an IHC score ≤4; SMYD3-positive was defined as an IHC score >4. **E** Kaplan-Meier analysis of overall survival in 148 patients with HCC stratified by SMYD3 expression. The median expression level was used as the cut-off. Low SMYD3 expression in each of the 148 patients was defined as a value below the 50th percentile. High SMYD3 expression in each of the 148 patients was defined as a value above the 50th percentile. **F**, **G** Expression of the SMYD3 mRNA and protein in different HCC cell lines. Bar = 200 μm. Data are presented as the means ± SD of three separate experiments, each performed in triplicate. *P < 0.05; **P < 0.01.

We first investigated the relative expression of SMYD3 in HCC tissues from 16 patients using reverse transcription-polymerase chain reaction (RT-PCR). Our analysis of SMYD3 mRNA levels revealed significantly higher expression in 75% (12/16) of HCC tissues than in matched noncancerous tissues (Fig. 1A). A subsequent quantitative real-time polymerase chain reaction (qRT-PCR) analysis of another cohort of 80 pairs of HCC samples confirmed this positive relationship (62.5%, 50/80) (Supplementary Fig. 1), and the expression levels of SMYD3 were remarkably higher in the tumor specimens than those in matched noncancerous tissues (Fig. 1B). We performed immunohistochemistry (IHC) on microarrays containing paired HCC and noncancerous tissues from 148 patients to discern the relationship between the expression of the SMYD3 protein and HCC. The IHC scores were in accordance with the staining intensity (range 0 to 12). Obviously, the SMYD3 protein was also expressed at high levels in HCC samples (Fig. 1C). Furthermore, patients with HCC overexpressing SMYD3 had more aggressive microvascular invasion (ratio: 43/94 vs. 15/54, *p* = 0.031) and a more advanced pTNM stage (ratio: 52/94 vs. 19/35, *p* = 0.018) than patients with low levels of SMYD3 (Fig. 1D). In addition, the Kaplan-Meier method showed that patients with high SMYD3 expression had a significantly worse prognosis than those with low SMYD3 expression (*p* = 0.02) (Fig. 1E). We detected SMYD3 expression in HCC cell lines using qRT-PCR and western blotting to elucidate the role of SMYD3 in HCC. SMYD3 was expressed at higher levels in multiple HCC cell lines (SK-Hep-1, Huh-7, PLC/PRF/5, Hep3B, SMMC-7721, and HepG2.2.15) than in LO2 cells (Fig. 1F, G and Supplementary Fig. 2).

### Overexpression of SMYD3 facilitated the proliferation, colony formation, and migration of HCC cells

**Fig. 2:**
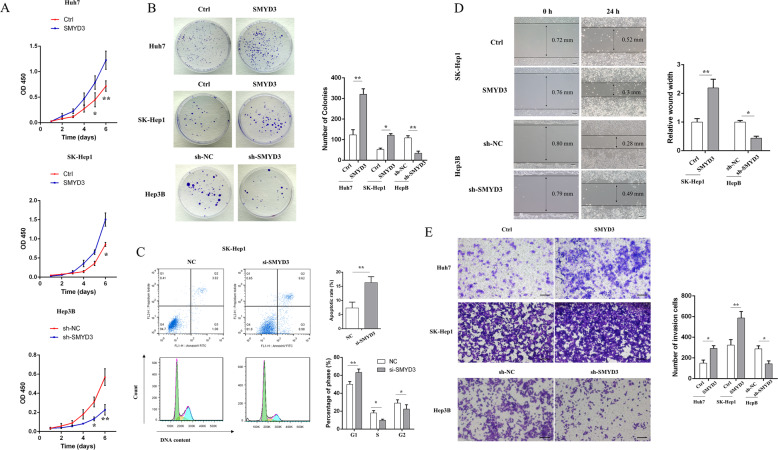
SMYD3 promoted the proliferation, colony formation, and migration of HCC cells. CCK8 (**A**), colony formation (**B**), cell cycle and apoptosis analysis using flow cytometry (**C**), wound-healing (**D**), and transwell assays (**E**) were performed to evaluate cell proliferation and migration in response to the overexpression or knockdown of SMYD3 in Huh7, SK-Hep1, and Hep3B cells. Bar = 100 μm. Data are presented as the means ± SD of three separate experiments, each performed in triplicate. *P < 0.05; **P < 0.01.

Overexpression of SMYD3 was induced by stably transfecting a plasmid containing the cDNA into cells with low SMYD3 expression (Huh-7 and SK-Hep-1), and knockdown of SMYD3 (sh-SMYD3) was achieved by stably transfecting an shRNA into Hep3B cells with high SMYD3 expression to evaluate the effects of SMYD3 on cell proliferation and migration. The cell lines with ectopic SMYD3 expression exhibited significantly increased cell growth, while cells with SMYD3 knockdown showed reduced cell growth (Fig. 2A). Colony formation assays validated the results described above; the number of colonies was obviously increased and decreased in the SMYD3 overexpression and knockdown groups, respectively, compared with the control group (Fig. 2B). A flow cytometry analysis was performed to evaluate whether SMYD3 affects HCC cells by altering the cell cycle profile and apoptosis. As shown in Fig. 2C, more cells were distributed in the G1 phase after SMYD3 knockdown, suggesting that knockdown of SMYD3 induced cell cycle arrest in the G1/S phase. In addition, apoptosis assays also revealed that knockdown of SMYD3 exerted an apoptosis-inducing effect on HCC cells. Moreover, wound-healing and transwell assays indicated that the upregulation of SMYD3 enhanced the migration capacities of SK-Hep1 cells, while the knockdown of SMYD3 markedly reduced the migration of Hep3B cells (Fig. 2D, E). Based on these results, SMYD3 overexpression plays an important role in promoting the proliferation, colony formation, and migration of HCC cells.

### SMYD3 potentially regulated the expression of S1PR1

**Fig. 3:**
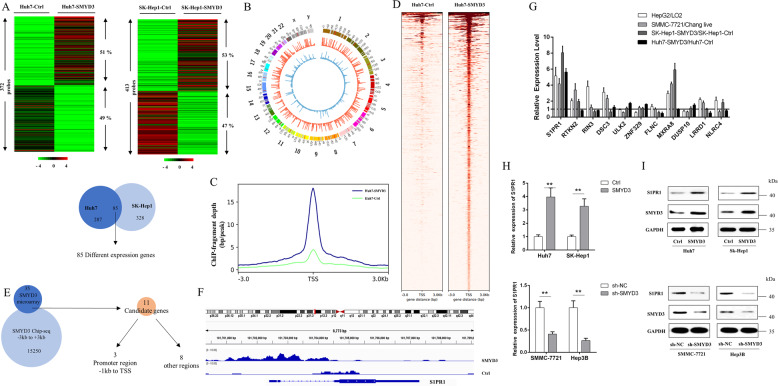
SMYD3 regulated the expression of S1PR1. **A** Upper panel, the heat map of differentially expressed mRNAs in Huh7-SMYD3 cells compared with Huh7-Ctrl cells and in SK-Hep1-SMYD3 compared SK-Hep1-Ctrl cells (≥ 2-fold). The red color represents higher expression levels, and the green color represents lower expression levels (log_2_ fold change). Lower panel, 85 candidate genes were selected after determining the overlap of the two datasets. **B** Circos plots showing genes on each of the 23 chromosomes with increased SMYD3 occupancy in ChIP-seq (Huh7-SMYD3 red compared with Huh7-Ctrl blue). **C** Profile plot showing the distribution of indicated ChIP-seq signals around the TSS regions in cells overexpressing SMYD3. **D** Heat map of SMYD3 ChIP-seq binding profiles around TSS regions (−3 kb to 3 kb) in Huh7-Ctrl and Huh7-SMYD3 cells. **E** Venn diagram showing the overlap of SMYD3-upregulated genes and genes with SMYD3 binding sites at TSS (−3 kb to 3 kb) regions. **F** IGV (Integrative Genomics Viewer) profile of SMYD3-enriched regions over the S1PR1 locus which was mainly at the promoter regions (−1 kb to the TSS). **G** mRNA expression levels of the 11 candidate genes in HepG2/LO2 (immortalized hepatocytes), SMMC-7721/Chang live (immortalized hepatocytes), Sk-Hep1-SMYD3/Sk-Hep1-Ctrl (transiently transfected with SMYD3), and Huh7-SMYD3/Huh7-Ctrl cells (transiently transfected with SMYD3). **H** qPCR and (**I**) western blot analyses of S1PR1 expression in HCC cells with stable SMYD3 overexpression (Huh7-Ctrl vs. Huh7-SMYD3 and SK-Hep1-Ctrl vs. SK-Hep1-SMYD3) or stable knockdown (SMMMC-7721-sh-NC vs. SMMMC-7721-sh-SMYD3 and Hep3B-sh-NC vs. Hep3B-sh-SMYD3). Data are presented as the means ± SD of three separate experiments, each performed in triplicate. *P < 0.05; **P < 0.01.

We analyzed the mRNA expression levels in Huh7-SMYD3 cells and SK-Hep1-SMYD3 cells and compared them with those in Huh7-Ctrl and SK-Hep1-Ctrl cells, respectively, by performing a microarray analysis to further explore the SMYD3-mediated mechanisms regulating the expression of downstream genes to promote oncogenesis. As shown in Fig. 3A, the microarray results revealed that 189 mRNAs were upregulated and 183 mRNAs were downregulated by SMYD3 in SK-Hep1 cells, and 219 mRNAs were upregulated and 194 mRNAs were downregulated by SMYD3 in Huh7 cells (≥2-fold). After intersecting the two datasets, 85 genes were selected for further study (Supplementary Table 1).

Next, we performed chromatin immunoprecipitation sequencing (ChIP-seq) to profile the genome-wide occupancy of SMYD3 in Huh7 cells transfected with Ctrl or SMYD3 vectors. As shown in Fig. 3B, the overall distribution of SMYD3 occupancy throughout the genome was increased upon the overexpression of SMYD3. Furthermore, SMYD3 binding peaks showed significant central enrichment and increased at their TSS regions (−3 kb to 3 kb) upon the overexpression of SMYD3 compared to Ctrl cells (Fig. 3C, D). SMYD3 binding was enriched around the TSSs of the target genes, which prompted us to analyze the SMYD3 coverage around the SMYD3 peak centers at −3 kb to the TSS and at the TSS to +3 kb. By comparing the upregulated gene identified from the microarray with the increased SMYD3 occupancy genes (−3 kb to 3 kb) from the ChIP-seq list, we identified 11 overlapping genes (Fig. 3E), and S1PR1, RIN3, and RTKN2 were located at the 1 kb promoter region (−1 kb to the TSS) (Fig. 3F, Supplementary Fig. 3 and 4). As shown in the ChIP landscape, SMYD3 binding peaks around the TSS regions of S1PR1 were higher in SMYD3-overexpressing cells. Then, we analyzed the relative mRNA expression levels of these 11 genes in HepG2/LO2, SMMC-7721/Chang live, Sk-Hep1-SMYD3/Sk-Hep1-Ctrl, and Huh7-SMYD3/Huh7-Ctrl cells. The expression levels of S1PR1, MXRA8, and RTKN2, especially S1PR1, were significantly upregulated in HCC and SMYD3-overexpressing cell lines (Fig. 3G).

Based on these results, we selected S1PR1 as the candidate gene for subsequent experiments. qRT-PCR and western blotting were performed to confirm whether SMYD3 regulates S1PR1 expression, and the results showed that SMYD3 overexpression upregulated the S1PR1 mRNA and protein, whereas knockdown of SMYD3 produced the opposite outcomes (Fig. 3H, I). These results strongly support the concept that S1PR1 is regulated by SMYD3 in HCC cells.

### SMYD3 regulated S1PR1 via histone lysine methylation of the S1PR1 promoter

**Fig. 4:**
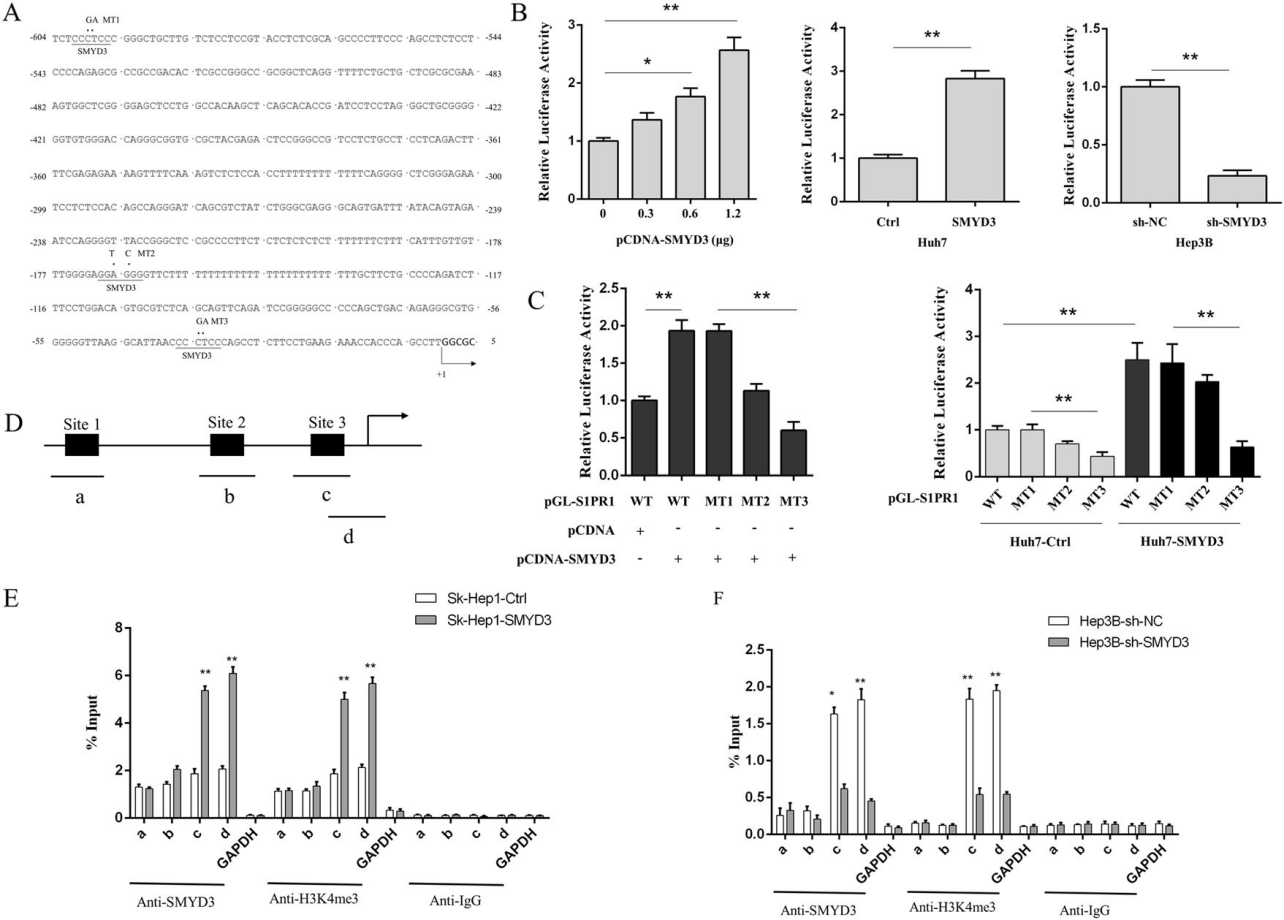
SMYD3 regulated S1PR1 via methylation of the S1PR1 promoter. **A** The specific SMYD3 DNA binding site (–CCCTCC-) in the S1PR1 promoter. **B** Luciferase assays with the S1PR1 promoter were performed in HCC cells transfected with different amounts of pCDNA-SMYD3 vector in SK-Hep1 cells or cells with stable overexpression (Huh7 cells) or knockdown (Hep3B cells) of SMYD3. **C** SK-Hep1 cells were cotransfected with pGL-S1PR1-WT, pGL-S1PR1-MT1, pGL-S1PR1-MT2, pGL-S1PR1-MT3, and SMYD3 (pcDNA3.1-SMYD3) (left panel) and Huh7-Ctrl and Huh7-SMYD3 cells were cotransfected with pGL-S1PR1-WT, MT1, MT2 and MT3 (right panel). The promoter activities were measured by performing luciferase reporter assays. **D** The examined primer position for ChIP-PCR assays at the S1PR1 locus was indicated. **E**, **F** The ChIP assay was performed in SK-Hep1-Ctrl and SK-Hep1-SMYD3 cells (**E**) and Hep3B-sh-NC and Hep3B-sh-SMYD3 (**F**) cells using specific antibodies. The immunoprecipitated DNA was measured with real-time PCR using the primers listed above. Data are presented as the means ± SD of three separate experiments, each performed in triplicate. *P < 0.05; **P < 0.01.

Based on previous studies showing that the SET domain of SMYD3, a histone lysine methyltransferase, affects downstream gene expression [20, 21], we hypothesized that SMYD3 meditated lysine methylation of the S1PR1 promoter sequence to regulate S1PR1 expression in HCC cells. We investigated the specific DNA sequence that bound the motifs of SMYD3 to verify this assumption and identified three –CCCTCC– sites in the S1PR1 promoter (Fig. 4A) [20, 22]. Luciferase assays revealed increased luciferase activity in SMYD3-transfected cells in a dose-dependent manner, while promoter activities decreased in response to SMYD3 knockdown in HCC cells (Fig. 4B). Then, we mutated the three SMYD3-binding sites via site-directed mutagenesis. Compared with the control wild-type sequence, mutations at sites 2 and 3 reduced the luciferase activity, especially when site 3 was mutated (Fig. 4C, left panel). Furthermore, Huh7 cells transfected with the SMYD3 overexpression vector showed increased luciferase activity, whereas the same cells with mutations in SMYD3-binding sites showed reduced activity (Fig. 4C, right panel). Moreover, we carried out ChIP-PCR with antibodies recognizing SMYD3 and H3K4me3 at the above 3 sites (Fig. 4D). As shown in Fig. 4E, the S1PR1 promoter was indeed occupied by the SMYD3 protein, and this binding was enhanced in the presence of ectopically expressed SMYD3. Correspondingly, knockdown of SMYD3 led to its downregulation at the S1PR1 promoter region (Fig. 4F). We concluded that SMYD3 indirectly affected S1PR1 transcription by meditating the methylation of the S1PR1 promoter at site 3 in HCC cells. These results support our hypothesis that SMYD3 regulates S1PR1 expression by meditating histone lysine methylation at the S1PR1 promoter.

### SMYD3 regulated the downstream signaling pathway of S1PR1

**Fig. 5:**
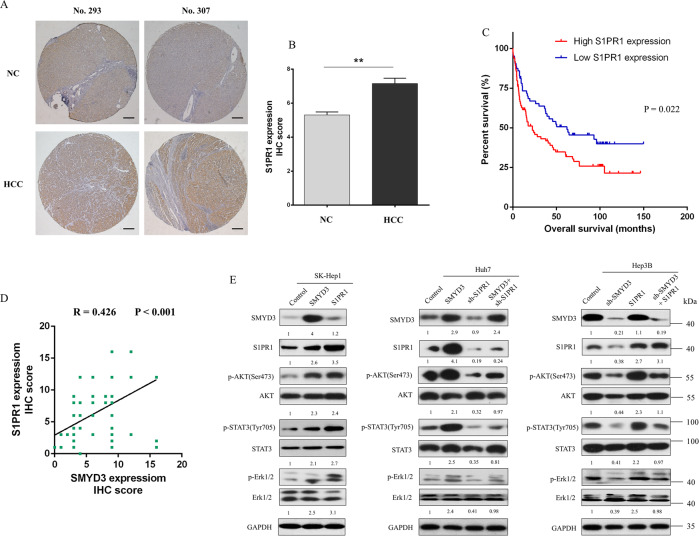
SMYD3 regulated its downstream signaling pathways via S1PR1. **A** IHC staining was performed on paired tumor and adjacent noncancerous tissues from 148 patients with HCC. **B** Statistical analysis of the IHC scores between HCC and adjacent normal tissues. **C** Kaplan-Meier analysis of overall survival in patients with HCC stratified according to the S1PR1 expression status. The median expression level of S1PR1 was used as the cut-off. **D** Clinical samples from the same set of tissues were analyzed in the two groups as indicated. The correlation between SMYD3 and S1PR1 expression was assessed by performing a linear regression analysis. **E** Western blot analysis of SMYD3, S1PR1, p-AKT, AKT, p-STAT3, STAT3, p-Erk1/2, and Erk1/2 expression levels in control cells and cells overexpressing S1PR1 and cells overexpressing SMYD3 combined with knockdown of SMYD3 or S1PR1. Images are representative of two experiments. Bar = 200 μm. *P < 0.05; **P < 0.01.

We performed IHC experiments on paired tissues from 148 patients with HCC to detect the differential expression of S1PR1 between HCC and adjacent normal tissues and found abundantly higher IHC scores in HCC tissues than in the paired normal tissues (Fig. 5A, B). Furthermore, higher S1PR1 expression was correlated with shorter survival (*p* = 0.022) (Fig. 5C). All these results support the hypothesis that S1PR1 overexpression indicates a poor prognosis for patients with HCC. Moreover, S1PR1 expression was positively correlated with SMYD3 expression, consistent with our previous results showing that S1PR1 is the downstream effector of SMYD3 (Fig. 5D).

Recent studies have suggested that the activation of S1PR1 is involved in regulating various aggressive biological phenotypes of tumors by modulating its downstream signaling pathways [23–25]. We investigated changes in the expression of constituents of those signaling pathways, including the AKT, STAT3, and MAPK pathways, using western blotting to validate whether the downstream signaling pathways of SMYD3 were regulated by S1PR1. Overexpression of SMYD3 or S1PR1 increased the levels of phosphorylated AKT, STAT3, and Erk1/2, while knockdown of SMYD3 or S1PR1 reduced p-AKT, p-STAT3, and p-Erk1/2 levels. Furthermore, knockdown of S1PR1 ablated the increases in the levels of p-AKT, p-STAT3, and p-Erk1/2 induced by SMYD3, and when cells with SMYD3 knockdown were transfected with an S1PR1-overexpressing plasmid, the decreases in p-AKT, p-STAT3, and p-Erk1/2 levels were rescued (Fig. 5E). Based on these results, SMYD3 increased AKT, STAT3, and Erk1/2 activation by promoting S1PR1 expression.

### SMYD3 promoted HCC progression through S1PR1

**Fig. 6:**
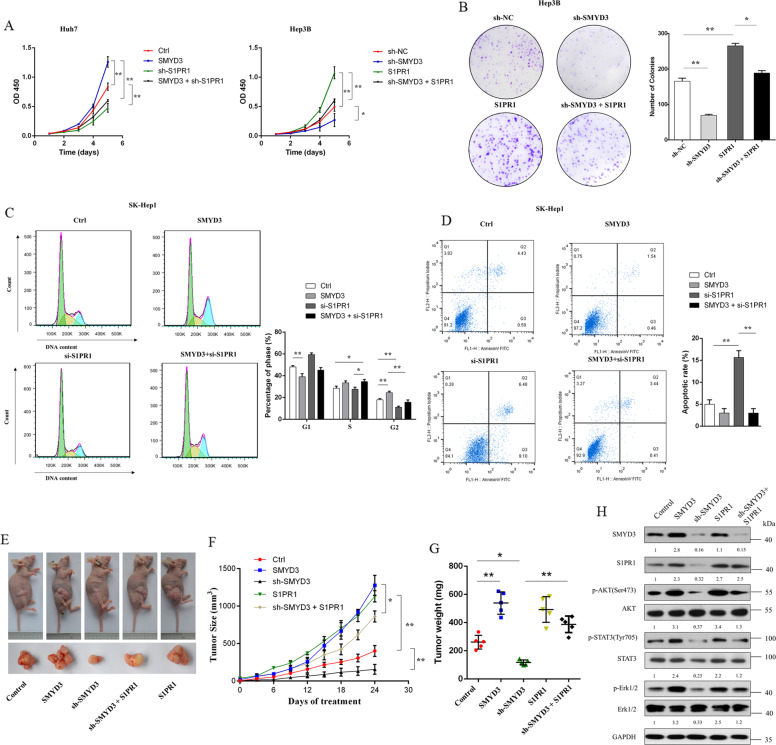
SMYD3 promoted HCC progression via S1PR1. CCK8 (**A**), colony formation (**B**), cell cycle (**C**), and apoptosis (**D**) assays were performed to evaluate cell proliferation and migration in response to the overexpression or knockdown of SMYD3 combined with the overexpression or knockdown of S1PR1 in Huh7, SK-Hep1, and Hep3B cells. Data are presented as the means ± SD of three separate experiments, each of which was performed in triplicate. **E** Nude mice were subcutaneously inoculated with Huh7 cells transfected with control, SMYD3 overexpression, SMYD3 shRNA, S1PR1 overexpression, and SMYD3 shRNA combined with S1PR1 overexpression vectors. Photographs show mice bearing subcutaneous tumors from each group (upper panel) or the dissected tumors (lower panel) (*n* = 5). **F** Tumor volumes were measured and recorded every 3 days, and tumor growth curves were created for each group. **G** The weight of the local tumors from each group was measured. **H** Western blots showing SMYD3, S1PR1, p-AKT, AKT, p-STAT3, STAT3, p-Erk1/2, and Erk1/2 expression levels in Huh7 tumors. Bar = 50 μm. The image is representative of two experiments. *P < 0.05; **P < 0.01.

We performed CCK8, colony formation, cell cycle, apoptosis, and transwell assays to explore whether SMYD3-mediated S1PR1 activity was involved in cancer cell development and progression. SMYD3 or S1PR1 was stably or transiently overexpressed or knocked down in HCC cells. The proliferation capacities of SMYD3-overexpressing cells were abrogated by S1PR1 knockdown (Fig. 6A, C and D). SMYD3 knockdown reduced the proliferation and migration capacities, but overexpression of S1PR1 in cells with SMYD3 knockdown reversed the effects compared to those in the control cells (Fig. 6A, B and Supplementary Fig. 5).

We assessed the effects of altered expression, including SMYD3 knockdown, SMYD3 overexpression, S1PR1 overexpression, and the combination of SMYD3 knockdown and S1PR1 overexpression, on the growth of local tumors in nude mice to test whether SMYD3 promotes HCC progression via S1PR1 in vivo (Fig. 6E). The SMYD3-overexpressing and S1PR1-overexpressing groups showed significantly increased tumor growth compared to the control group. In contrast, SMYD3 knockdown significantly suppressed tumor growth and reduced tumor proliferation; these effects were largely reversed by S1PR1 overexpression (Fig. 6F, G). In addition, the western blot results from excised tumors showed that SMYD3 regulates AKT, STAT3, and MAPK signaling pathways through S1PR1 in vivo (Fig. 6H). Our experiments revealed that S1PR1 is a downstream gene of SMYD3 and that SMYD3 regulates the expression of S1PR1 to facilitate carcinogenesis in HCC cells.

## Discussion

SMYD3 was originally described as an H3K4 methyltransferase that regulates the transcriptional activities of downstream genes involved in cancer progression [20]. Furthermore, a large amount of evidence indicates critical roles for SMYD3 in the proliferation, invasion, and migration of different tumor cells [13, 14, 26–28]. We investigated venous invasion, pTNM stage, and overall survival in HCC samples from a SMYD-positive group and SMYD3-negative group to confirm the correlations between SMYD3 expression and the clinical characteristics and pathological parameters in patients with HCC. The results confirmed that SMYD3 overexpression is a risk factor in patients with HCC, consistent with other studies [29–31]. We established SMYD3-overexpressing and SMYD3-knockdown vectors and transfected them into HCC cells to investigate changes in biological phenotypes, such as cell proliferation, migration, and colony formation. SMYD3 dramatically facilitated the development and progression of HCC, and we concluded that SMYD3 is involved in the aggressive behaviors of HCC and plays a crucial role in determining the prognosis of patients.

SMYD3 methylates not only histone H3 at lysine 4 but also histone H3 at lysine 9, histone H4 at lysine 5, and histone H4 at lysine 20 [13, 18, 32]; these histone lysine methylation events meditated by the SET domain of SMYD3 exert significant effects on cancer development and progression. As shown in previous studies, aberrant histone lysine methylation, including monomethylation, dimethylation, and trimethylation, is often associated with promoters and enhancers of oncogenes in cancer cells [33], which provides strong evidence supporting the hypothesis that SMYD3 promotes carcinogenesis via histone lysine methylation. In addition, the SET domain has been shown to catalyze the trimethylation of H3K4, which is associated with increased transcription of target genes [34, 35]. Based on these studies, we used ChIP-seq to investigate the genes that are highly overexpressed in HCC and combined those results with SMYD3 target genes to explore whether H3K4me3 of downstream genes is a mechanism by which SMYD3 promotes the carcinogenesis of HCC. From the identified genes, we selected S1PR1 for further study due to its high expression in HCC.

Some studies have reported a critical role for S1PR1 in cancer biology [23, 36, 37]. However, the mechanism by which SMYD3 mediates S1PR1 expression has not been elucidated. We verified that the expression and function of S1PR1 in HCC were regulated by SMYD3. The results implied that S1PR1 is the downstream gene of SMYD3. Furthermore, ChIP-PCR indicated that a critical site in the S1PR1 promoter exhibited a high level of H3K4me3 when targeted by SMYD3. At the same time, luciferase assays revealed that mutating these sites reduced the activity of SYMD3. Therefore, SMYD3 methylates lysines in histones at the S1PR1 promoter to regulate S1PR1 expression.

We performed western blotting to detect the levels of p-AKT, p-STAT3, and p-Erk1/2 in different treatment groups compared with those in the control group and to determine whether the downstream signaling pathways of S1PR1 are regulated by SMYD3. As expected, signaling pathways mediated by S1PR1 were upregulated by SMYD3 overexpression, whereas SMYD3 knockdown downregulated their levels. In vivo experiments were conducted to compare the tumor size in the treatment groups and the control group. According to the results, S1PR1 is involved in SMYD3-related HCC progression.

Collectively, these studies support the hypothesis that SMYD3 plays a crucial function in HCC progression, partially by mediating histone methylation at the downstream gene S1PR1, which affects critical signaling pathways associated with the carcinogenesis and progression of HCC.

## Patients and methods

### Patient samples

Eighty fresh HCC tissues and corresponding adjacent noncancerous liver tissues (more than 2 cm from the margin) were obtained from patients undergoing liver resection at Sun-Yat-Sen Memorial Hospital of Sun-Yat-Sen University between January 2008 and December 2012. Another 148 pairs of paraffin-embedded HCC samples and adjacent noncancerous tissues were obtained from January 1999 to December 2007. Eleven fresh normal liver samples and 10 paraffin-embedded normal liver samples were obtained from the specimen bank of the Department of Hepatobiliary Surgery at Sun-Yat-Sen Memorial Hospital. The use of clinical specimens was approved by the Ethical Institutional Review Board of the Sun-Yat-Sen Memorial Hospital.

### HCC cell lines

HEK293T cells and the hepatoma cell lines HepG2, Huh7, SK-hep1, PLC/PRF/5 (derived from HBV-infected liver), and Hep3B (derived from HBV-infected liver) were purchased from the Chinese Academy of Sciences Cell Bank of Type Culture Collection (CBTCCCAS). LO2, SMMC-7721, and HepG2.2.15 (HepG2-derivative with the integration of the HBV genome) cells [38, 39] were purchased from BioHermes. All cell lines had guaranteed authenticity through short tandem repeat profiling and comparison to DNA profiles of known cell lines. Cells were cultured in high-glucose Dulbecco’s modified Eagle’s medium (DMEM) supplemented with 10% fetal bovine serum (FBS) and incubated at 37 °C in an atmosphere containing 5% CO_2_.

### RNA extraction, reverse transcription, and quantitative real-time polymerase chain reaction (qRT-PCR)

Total RNA was extracted using TRIzol reagent according to the manufacturer’s instructions (Invitrogen, USA). Complementary DNA was synthesized from total RNA using reverse transcriptase and random primers. Quantitative real-time PCR was performed in the GoTaq® qPCR System (Promega, Beijing, China). The gene-specific primers are as follows: SMYD3, F 5′-GTCTTCAAACTTATGGATGGAGC-3′, 5′-GGCATCCTGTATTTCTTCTCTCA-3′; S1PR1, F 5′-CAGCAAATCGGACAATTCCT-3′; R 5′-GCCAGCGACCAAGTAAAGAG-3′; β-actin, F 5′-CTCCCTGGAGAAGAGCTACG-3′, R 5′-ACAGGACTCCATGCCCAG-3′; and GAPDH, F 5′-GAAGGTGAAGGTCGGAGTCAACG-3′; R 5′-TGCCATGGGTGGAATCATATTGG-3′.

### Construction of tissue microarrays and IHC

Tissue microarray blocks consisting of 148 paired HCC samples and 10 normal liver samples were constructed using a tissue microarray (Quick-Ray, UNITMA). IHC was performed to detect the expression of the SMYD3 and S1PR1 proteins in the tissue microarray blocks using IHC kits (Biohao Biotechnology, Guangzhou). The immunostaining was assessed by counting the total and positively stained cells at a magnification of ×40 in at least randomly selected 10 fields. The staining intensity and extent of the stained area were graded using the semiquantitative scoring system described below. For the staining intensity of the cytoplasm, no staining = 0; weak staining = 1; moderate staining = 2; and strong staining = 3; for the extent of stained cells, 0% = 0, 1 to 20% = 1, 21 to 50% = 2, 51 to 80% = 3, and 81 to 100% = 4. The final immunoreactive score was determined by multiplying the intensity score by the extent of stained cells (range 0 to 12) [40].

### Stable overexpression or knockdown of SMYD3 and S1PR1 in HCC Cells

The open reading frames of SMYD3 and S1PR1 were cloned into the retroviral vector pMSCV-eGFP-Puro according to the manufacturer’s instructions. The shRNAs targeting SMYD3 and S1PR1 were inserted into the lentiviral vector pGC-eGFP-Hygromycin (GV544); both constructs were generated by Genechem Company. The shRNA sequences of SMYD3 and S1PR1 are as follows: sh-SMYD3, 5′-GGATGGAGCACCTTCAGAATC-3′ and sh-S1PR1, 5′-CTGCTCAAGACCGTAATTAT-3′. Production and purification of the lentivirus and construction of the stably infected cell lines were performed as previously described [41].

### Proliferation assay

The MTT assay was conducted using a cell proliferation kit according to the manufacturer’s instructions. Briefly, 400 HCC cells in 200 μl of complete culture medium were added to each well of a 96-well plate and cultured at 37 °C in an atmosphere containing 5% CO_2_. Culture medium alone served as the negative control. Then, the MTT solution was added 24, 48, 72, 96, 120, and 144 h after seeding. The culture medium was removed, and 150 μl of DMSO was added and incubated with shaking for 4 h. The optical density value was detected at 490 nm, and a cell growth curve was drawn. HCC cells were divided into six pairs and tested three times.

### Flow cytometry assay

A total of 1 × 10^6^ cells were seeded into 6-well plates in a DMEM medium. The cells were harvested and stained with annexin V-FITC and propidium iodide (PI) 48 h later according to the manufacturer’s instructions. The cellular apoptotic rates were evaluated using a FACS Verse™ flow cytometer (Becton Dickinson, CA, USA). Cells for cell cycle analysis were resuspended in 200 ml PBS, fixed with 70% ice-cold ethanol overnight, and stained with PI. The cell growth phase was detected by the FACSVerse™ flow cytometer.

### Cell migration assay

The wound-healing test was conducted as follows: 5 × 10^5^ cells in high-glucose DMEM supplemented with 10% FBS were seeded into 6-well plates and incubated overnight at 37 °C in an atmosphere containing 5% CO_2_. Pipette tips were used to scratch the monolayers, which were then washed with phosphate-buffered saline. A serum-free culture medium was added and incubated for another 48 h. Images were recorded at 0, 12, and 24 h. Transwell assays were conducted as follows: 1 × 10^5^ cells were suspended in a serum-free culture medium and added to the upper chamber of a transwell cell migration apparatus (Corning). The lower chamber contained a culture medium supplemented with 10% FBS. Cells were incubated for 24 h at 37 °C in an atmosphere containing 5% CO_2_. Cells that migrated through the membrane pores to the lower surface of the membrane were fixed with methanol and stained with crystal violet. Then, the cells were visualized and counted under a microscope.

### Colony formation assay

The colony formation assay was conducted as follows: 1 × 10^3^ HCC cells stably transfected with SMYD3 or control vectors were seeded into 35-mm dishes and cultured at 37 °C in a humidified atmosphere containing 5% CO_2_ for 2-3 weeks until colonies formed. Then, colonies were fixed with polymerized formaldehyde and visualized by performing crystal violet staining. Colonies containing more than 10 cells were counted under a microscope. All conditions were analyzed in triplicated and independently tested three times.

### Western blot analysis

Cells were lysed, and proteins were collected and separated by SDS-PAGE on 10% gels. The separated proteins were then transferred onto polyvinylidene fluoride membranes (Millipore), blocked with TBST containing 5% nonfat dry milk for 2 h, and incubated with antibodies against SMYD3, S1PR1, Erk, p-Erk, GAPDH (all from Santa Cruz Biotechnology) AKT, p-AKT, Stat3, and p-Stat3 (Cell Signaling Technology) overnight at 4 °C. The GAPDH antibody was used as an internal control. Then, membranes were washed 3 times with TBST and incubated with a horseradish peroxidase-conjugated secondary antibody (Biohao Biotechnology) before the protein bands were visualized with an ECL plus western blot detection kit (Beyotime Biotechnology). Quantitative increases in protein phosphorylation were evaluated by performing a densitometry analysis of the ratio of phosphorylated protein/total protein of the treated cells. All western blots were repeated 2–3 times, and the mean changes in the treated groups compared with the non-treated cells are shown under the gels in the figures.

### Microarray analysis

A cell mRNA microarray was constructed according to the instructions from Affymetrix Inc. and was analyzed at the Gene ChIP Analysis Center at Sun Yat-Sen Memorial Hospital of Sun Yat-Sen University. Changes greater than two-fold compared to the control were considered significant.

### Luciferase assays

The 5′-flanking region (nucleotides −604 to +5) of S1PR1 was cloned into the pGL3-Basic vector (Promega) by Generay Biotech. The mutant constructs MT1, MT2, and MT3 were generated using a Site-Directed Mutagenesis Kit (Clontech) and sequences were verified. The wild-type and mutant fragments were inserted into a pmirGLO Dual-Luciferase vector (Promega) downstream of the luciferase gene, and the resulting constructs were designated pGL-S1PR1-WT, pGL-S1PR1-MT1, pGL-S1PR1-MT2, and pGL-S1PR1-MT3. Cells were infected with the indicated vector and then transfected with the indicated luciferase constructs, as described in the corresponding figure legend. Cell transfection was performed using Lipofectamine 2000 (Invitrogen) according to the manufacturer’s instructions. According to the manufacturer’s protocol, the cells were assayed 24 h after transfection to determine both firefly and Renilla luciferase activities using a luciferase assay kit (Promega). All transfections were performed in triplicate.

### Chromatin immunoprecipitation

The cells were grown in three 100-mm culture dishes to 80% confluence. Then, they were fixed with formaldehyde, and the ChIP protocol was performed using a SimpleChiP^TM^ Enzymatic Chromatin IP Kit (Agarose Beads) from Cell Signaling Technology according to their instructions. For sonication, 14 cycles of 30 s ON and 30 s OFF were conducted to obtain the required fragment sizes. The ChIP products were analyzed by Ribobio for ChIP-seq or PCR analysis for ChIP-PCR. RNA-seq data are deposited in Sequence Read Archive (SRA) database and are available through the accessions PRJNA699769. The primers for the S1PR1 promoter are listed below: a, F 5′-CAGTTGCGAGTAGCACGAGG-3′, R 5′-AGGTACGGAGGAGACAAGCAG-3′; b, F 5′-GGCGAGGGCAGTGATTTAT-3′, R 5′-CTGAACTGCTGAGACGCACT-3′; c, F 5′-CTTTCCTGGACAGTGCGTCT-3′, R 5′-CCAGTTCCCTGCCTGCTAC-3′; and d, F 5′-CCTCCCAGCCTTCCTGAA-3′, R 5′-CACTCCAATGGCCAGTCC-3′. All experiments were performed at least three times.

### Animal studies

For the tumor formation assay, 4-week-old to 5-week-old BALB/c athymic nude mice were used and randomly assigned to individual experimental groups. The animal care and experimental protocols were approved by the Animal Research Committee of Sun Yat-sen University. The animal experiments were performed in accordance with established guidelines. In total, 1 × 10^6^ cells were suspended in 200 μl of serum-free DMEM and injected into the right flanks of the mice. The tumor volume was measured every 3 days using calipers. The tumor volume was calculated using the following formula: *V* = (width^2^ × length) × 0.5. The tumor samples were collected from the mice, and protein expression was analyzed using western blotting.

### Statistical analysis

Statistical analyses were performed using SPSS version 19.0 software. Continuous data are reported as the means ± SD, and the significance of differences was compared using Student’s *t*-tests, Fisher’s exact tests, chi-square tests and one-way ANOVA with Bonferroni post hoc corrections, as appropriate. The cumulative survival probability was evaluated using the Kaplan-Meier method, and differences were assessed using log-rank tests. *P* < 0.05 was considered statistically significant.

## Supplementary information

Supplemental data

Supplemental Figure 1

Supplemental Figure 2

Supplemental Figure 3

Supplemental Figure 4

Supplemental Figure 5

## References

[1] National Cancer Institute. Surveillance Research Program, National Cancer Institute. Fast stats: an interactive tool for access to SEER cancer statistics. http://surveillance.cancer.gov/. April 15, 2020.

[2] Alqahtani A, Khan Z, Alloghbi A, Said Ahmed TS, Ashraf M, Hammouda DM. Hepatocellular carcinoma: molecular mechanisms and targeted therapies. Medicina. 2019;55:526. 10.3390/medicina55090526 PMC6780754 31450841

[3] Huang B, Tian ZF, Li LF, Fan Y, Yin HY, Li Y (2019). LHX3 is an advanced-stage prognostic biomarker and metastatic oncogene in hepatocellular carcinoma. Cancer Biomark.

[4] Huang P, Xu Q, Yan Y, Lu Y, Hu Z, Ou B (2020). HBx/ERalpha complex-mediated LINC01352 downregulation promotes HBV-related hepatocellular carcinoma via the miR-135b-APC axis. Oncogene.

[5] Mak LY, Wong DK, Pollicino T, Raimondo G, Hollinger FB, Yuen MF (2020). Occult hepatitis B infection and hepatocellular carcinoma: Epidemiology, virology, hepatocarcinogenesis and clinical significance. J Hepatol.

[6] Vieira FQ, Costa-Pinheiro P, Almeida-Rios D, Graça I, Monteiro-Reis S, Simões-Sousa S (2015). SMYD3 contributes to a more aggressive phenotype of prostate cancer and targets Cyclin D2 through H4K20me3. Oncotarget.

[7] Fenizia C, Bottino C, Corbetta S, Fittipaldi R, Floris P, Gaudenzi G (2019). SMYD3 promotes the epithelial-mesenchymal transition in breast cancer. Nucleic Acids Res.

[8] Li B, Pan R, Zhou C, Dai J, Mao Y, Chen M (2018). SMYD3 promoter hypomethylation is associated with the risk of colorectal cancer. Future Oncol.

[9] Jiang Y, Lyu T, Che X, Jia N, Li Q, Feng W (2019). Overexpression of SMYD3 in ovarian cancer is associated with ovarian cancer proliferation and apoptosis via methylating H3K4 and H4K20. J Cancer.

[10] Sarris ME, Moulos P, Haroniti A, Giakountis A, Talianidis I (2016). Smyd3 is a transcriptional potentiator of multiple cancer-promoting genes and required for liver and colon cancer development. Cancer Cell.

[11] Tracy C, Warren JS, Szulik M, Wang L, Garcia J, Makaju A (2018). The Smyd family of Methyltransferases: role in cardiac and skeletal muscle physiology and pathology. Curr Opin Physiol.

[12] Sirinupong N, Brunzelle J, Doko E, Yang Z (2011). Structural insights into the autoinhibition and posttranslational activation of histone methyltransferase SmyD3. J Mol Biol.

[13] Chen LB, Xu JY, Yang Z, Wang GB (2007). Silencing SMYD3 in hepatoma demethylates RIZI promoter induces apoptosis and inhibits cell proliferation and migration. World J Gastroenterol.

[14] Wang SZ, Luo XG, Shen J, Zou JN, Lu YH, Xi T (2008). Knockdown of SMYD3 by RNA interference inhibits cervical carcinoma cell growth and invasion in vitro. BMB Rep.

[15] Zhou Z, Jiang H, Tu K, Yu W, Zhang J, Hu Z (2019). ANKHD1 is required for SMYD3 to promote tumor metastasis in hepatocellular carcinoma. J Exp Clin Cancer Res.

[16] Mazur PK, Reynoird N, Khatri P, Jansen PW, Wilkinson AW, Liu S (2014). SMYD3 links lysine methylation of MAP3K2 to Ras-driven cancer. Nature.

[17] Yoshioka Y, Suzuki T, Matsuo Y, Nakakido M, Tsurita G, Simone C (2016). SMYD3-mediated lysine methylation in the PH domain is critical for activation of AKT1. Oncotarget.

[18] Van Aller GS, Reynoird N, Barbash O, Huddleston M, Liu S, Zmoos AF (2012). Smyd3 regulates cancer cell phenotypes and catalyzes histone H4 lysine 5 methylation. Epigenetics.

[19] Tsai CH, Chen YJ, Yu CJ, Tzeng SR, Wu IC, Kuo WH (2016). SMYD3-mediated H2A.Z.1 methylation promotes cell cycle and cancer proliferation. Cancer Res.

[20] Hamamoto R, Furukawa Y, Morita M, Iimura Y, Silva FP, Li M (2004). SMYD3 encodes a histone methyltransferase involved in the proliferation of cancer cells. Nat Cell Biol.

[21] Cock-Rada AM, Medjkane S, Janski N, Yousfi N, Perichon M, Chaussepied M (2012). SMYD3 promotes cancer invasion by epigenetic upregulation of the metalloproteinase MMP-9. Cancer Res.

[22] Liu C, Fang X, Ge Z, Jalink M, Kyo S, Björkholm M (2007). The telomerase reverse transcriptase (hTERT) gene is a direct target of the histone methyltransferase SMYD3. Cancer Res.

[23] Liu Y, Zhi Y, Song H, Zong M, Yi J, Mao G (2019). S1PR1 promotes proliferation and inhibits apoptosis of esophageal squamous cell carcinoma through activating STAT3 pathway. J Exp Clin Cancer Res.

[24] Zhou P, Huang G, Zhao Y, Zhong D, Xu Z, Zeng Y (2014). MicroRNA-363-mediated downregulation of S1PR1 suppresses the proliferation of hepatocellular carcinoma cells. Cell Signal.

[25] Go H, Kim PJ, Jeon YK, Cho YM, Kim K, Park BH (2015). Sphingosine-1-phosphate receptor 1 (S1PR1) expression in non-muscle invasive urothelial carcinoma: association with poor clinical outcome and potential therapeutic target. Eur J Cancer.

[26] Hamamoto R, Silva FP, Tsuge M, Nishidate T, Katagiri T, Nakamura Y (2006). Enhanced SMYD3 expression is essential for the growth of breast cancer cells. Cancer Sci.

[27] Wang L, Wang QT, Liu YP, Dong QQ, Hu HJ, Miao Z (2017). ATM signaling pathway is implicated in the SMYD3-mediated proliferation and migration of gastric cancer cells. J Gastric Cancer.

[28] Zhu Y, Zhu MX, Zhang XD, Xu XE, Wu ZY, Liao LD (2016). SMYD3 stimulates EZR and LOXL2 transcription to enhance proliferation, migration, and invasion in esophageal squamous cell carcinoma. Hum Pathol.

[29] Wang Y, Xie BH, Lin WH, Huang YH, Ni JY, Hu J (2019). Amplification of SMYD3 promotes tumorigenicity and intrahepatic metastasis of hepatocellular carcinoma via upregulation of CDK2 and MMP2. Oncogene.

[30] Fei X, Ma Y, Liu X, Meng Z (2017). Overexpression of SMYD3 is predictive of unfavorable prognosis in hepatocellular carcinoma. Tohoku J Exp Med.

[31] Li RD, Tang YH, Wang HL, Yang D, Sun LJ, Li W (2018). The SMYD3 VNTR 3/3 polymorphism confers an increased risk and poor prognosis of hepatocellular carcinoma in a Chinese population. Pathol Res Pract.

[32] Foreman KW, Brown M, Park F, Emtage S, Harriss J, Das C (2011). Structural and functional profiling of the human histone methyltransferase SMYD3. PLoS ONE.

[33] Heintzman ND, Stuart RK, Hon G, Fu Y, Ching CW, Hawkins RD (2007). Distinct and predictive chromatin signatures of transcriptional promoters and enhancers in the human genome. Nat Genet.

[34] Bernstein BE, Humphrey EL, Erlich RL, Schneider R, Bouman P, Liu JS (2002). Methylation of histone H3 Lys 4 in coding regions of active genes. Proc Natl Acad Sci USA.

[35] Strahl BD, Ohba R, Cook RG, Allis CD (1999). Methylation of histone H3 at lysine 4 is highly conserved and correlates with transcriptionally active nuclei in Tetrahymena. Proc Natl Acad Sci USA.

[36] Weichand B, Popp R, Dziumbla S, Mora J, Strack E, Elwakeel E (2017). S1PR1 on tumor-associated macrophages promotes lymphangiogenesis and metastasis via NLRP3/IL-1β. The. J Exp Med.

[37] Lin Q, Ren L, Jian M, Xu P, Li J, Zheng P (2019). The mechanism of the premetastatic niche facilitating colorectal cancer liver metastasis generated from myeloid-derived suppressor cells induced by the S1PR1-STAT3 signaling pathway. Cell Death Dis.

[38] Delaney WE, Isom HC (1998). Hepatitis B virus replication in human HepG2 cells mediated by hepatitis B virus recombinant baculovirus. Hepatology..

[39] Sells MA, Chen ML, Acs G (1987). Production of hepatitis B virus particles in Hep G2 cells transfected with cloned hepatitis B virus DNA. Proc Natl Acad Sci USA.

[40] Barnes DM, Harris WH, Smith P, Millis RR, Rubens RD (1996). Immunohistochemical determination of oestrogen receptor: comparison of different methods of assessment of staining and correlation with clinical outcome of breast cancer patients. Br J Cancer.

[41] Huang P, Zhuang B, Zhang H, Yan H, Xiao Z, Li W (2015). Hepatitis B Virus X Protein (HBx) is responsible for resistance to targeted therapies in hepatocellular carcinoma: ex vivo culture evidence. Clin. Cancer Res..

