# Correction: SMYD3 promotes hepatocellular carcinoma progression by methylating S1PR1 promoters

**DOI:** 10.1038/s41419-021-04071-2

**Published:** 2021-08-16

**Authors:** Heyun Zhang, Zhangyu Zheng, Rongqin Zhang, Yongcong Yan, Yaorong Peng, Hua Ye, Lehang Lin, Junyao Xu, Wenbin Li, Pinbo Huang

**Affiliations:** 1 Department of Hepatobiliary Surgery, Sun Yat-sen Memorial Hospital, Sun Yat-sen University, 510120 Guangzhou, China; 2 Guangdong Provincial Key Laboratory of Malignant Tumor Epigenetics and Gene Regulation, Medical Research Center, Sun Yat-sen Memorial Hospital, Sun Yat-sen University, 510120 Guangzhou, China; 3 Department of Nuclear Medicine, The Sixth Affiliated Hospital, Sun Yat-sen University, 510655 Guangzhou, China; 4 Department of Nuclear Medicine, Sun Yat-sen University Cancer Center, State Key Laboratory of Oncology in South China, Collaborative Innovation Center for Cancer Medicine, Guangzhou, China; 5 Department of Pancreaticobiliary Surgery, Sun Yat-sen Memorial Hospital, Sun Yat-sen University, 510120 Guangzhou, China; 6 Department of Preventive Health Care, Sun Yat-sen Memorial Hospital, Sun Yat-sen University, 510120 Guangzhou, China

**Keywords:** Liver cancer, Oncogenes

Correction to: *Cell Death and Disease* 10.1038/s41419-021-04009-8, published online 23 July 2021

The original version of this article unfortunately contained a mistake. Due to a typesetting error the following sentence was omitted: “These authors contributed equally: Heyun Zhang, Zhangyu Zheng, Rongqin Zhang”. We apologize for the error. The original article has been corrected.

