# Supplementary material for: SMYD3 promotes hepatocellular carcinoma progression by methylating S1PR1 promoters
Source: Cell Death Dis. 2021 Jul 23;12(8):731. doi: 10.1038/s41419-021-04009-8 (PMC8302584; doi:10.1038/s41419-021-04009-8)
Supplement: Supplementary file 1 — Supplemental data [file 41419_2021_4009_MOESM1_ESM.docx]

**Supplementary Table 1: Array data for differentially expressed mRNAs associated with SMYD3.**

| **GenBank Accession** | **Gene Symbol** | **Fold Change (SMYD3 vs Ctrl)** | | **Regulation** |
| --- | --- | --- | --- | --- |
|  |  | **Huh7** | **PLC/PRF/5** |  |
| NM_032825 | ZNF382 | 2.144211 | 11.3595439 | Up |
| AK090893 | ZNF329 | 2.8280766 | 10.7898358 | Up |
| NR_033266 | WASH5P | 3.2068925 | 3.8837679 | Up |
| NM_014683 | ULK2 | 2.2530093 | 7.9879577 | Up |
| NM_003355 | UCP2 | 3.7287545 | 23.9734295 | Up |
| NM_006074 | TRIM22 | 2.8934 | 11.7695536 | Up |
| NM_178545 | TMEM52 | 2.0890301 | 2.1839755 | Up |
| NM_003247 | THBS2 | 2.2022502 | 2.2980882 | Up |
| NM_013453 | SPANXA1 | 2.0272106 | 2.0887815 | Up |
| NM_007231 | SLC6A14 | 2.0703097 | 2.6840177 | Up |
| NM_014585 | SLC40A1 | 2.310186 | 3.4794686 | Up |
| NM_001400 | S1PR1 | 4.1451052 | 186.3671919 | Up |
| NM_001282941 | RTKN2 | 3.0660497 | 3.219306 | Up |
| NM_019055 | ROBO4 | 2.0011991 | 27.2430703 | Up |
| NM_002934 | RNASE2 | 2.6522122 | 4.1161961 | Up |
| NM_024832 | RIN3 | 3.7336358 | 3.815099 | Up |
| NM_005398 | PPP1R3C | 3.2133985 | 2.6490021 | Up |
| NR_003242 | PFN1P2 | 2.366323 | 2.3345656 | Up |
| NM_021209 | NLRC4 | 3.9336276 | 3.0566835 | Up |
| NM_032348 | MXRA8 | 3.5248125 | 8.8621423 | Up |
| NM_002754 | MAPK13 | 2.4153636 | 5.4678679 | Up |
| AK091323 | LRRD1 | 2.8199407 | 3.1846736 | Up |
| XM_003960901 | LOC101060085 | 2.1008694 | 2.8022602 | Up |
| AK125613 | LOC100131820 | 2.3973682 | 2.3016419 | Up |
| NM_002279 | KRT33B | 2.2384585 | 3.4278914 | Up |
| NM_001190981 | IL6ST | 2.0652099 | 2.2578357 | Up |
| NM_001191323 | GREM1 | 2.2333628 | 4.6743174 | Up |
| NM_015597 | GPSM1 | 2.3849663 | 2.3391883 | Up |
| DQ438879 | GLIS3 | 2.1984547 | 5.5538715 | Up |
| NM_000823 | GHRHR | 2.4531322 | 2.0867662 | Up |
| NM_001458 | FLNC | 2.7859095 | 46.0588377 | Up |
| NM_032130 | FAM186B | 2.0937468 | 4.8141811 | Up |
| NM_173544 | FAM129C | 2.6024847 | 2.1965027 | Up |
| NM_003633 | ENC1 | 2.7953346 | 55.5302544 | Up |
| NM_007207 | DUSP10 | 2.5789714 | 5.6424628 | Up |
| NM_024423 | DSC3 | 5.0900294 | 100.8114223 | Up |
| NM_014395 | DAPP1 | 2.4071486 | 2.8625423 | Up |
| XM_005251093 | CPNE3 | 2.235056 | 2.7292227 | Up |
| AK098702 | COL20A1 | 4.3886563 | 2.9173215 | Up |
| NM_024111 | CHAC1 | 2.0411798 | 2.4539713 | Up |
| NM_001038707 | CDC42SE1 | 2.873974 | 3.1529406 | Up |
| NM_030925 | CAB39L | 2.060337 | 2.0710968 | Up |
| NM_001007090 | C8orf48 | 5.4811446 | 12.1042946 | Up |
| NR_026865 | C7orf13 | 2.9083756 | 5.6299262 | Up |
| NM_032413 | C15orf48 | 2.9112243 | 70.0868364 | Up |
| NM_001125 | ADPRH | 2.0968283 | 4.9685886 | Up |
| NM_016378 | VCX2 | 2.7159372 | 3.6706341 | Down |
| NM_033199 | UCN2 | 2.726656 | 8.4677343 | Down |
| NR_026963 | TTC28-AS1 | 2.2569319 | 4.0810319 | Down |
| NM_173553 | TRIML2 | 2.1985394 | 3.8184152 | Down |
| NM_001164407 | TLCD2 | 3.0520212 | 2.4215519 | Down |
| AK122959 | TAS1R3 | 2.1495416 | 20.3411311 | Down |
| NM_003153 | STAT6 | 2.2269289 | 3.6681179 | Down |
| NM_174934 | SCN4B | 4.8498428 | 5.5369773 | Down |
| NM_001242359 | RHOBTB1 | 2.0571486 | 5.5924157 | Down |
| NM_006604 | RFPL3 | 3.5239337 | 2.8890074 | Down |
| NM_000962 | PTGS1 | 2.7809286 | 36.5498454 | Down |
| NM_214710 | PRSS57 | 3.0933177 | 2.5286268 | Down |
| NM_001004058 | OR8K5 | 2.0064073 | 4.5080224 | Down |
| NM_002514 | NOV | 5.8245678 | 30.9507829 | Down |
| NM_178493 | NOTUM | 3.3871944 | 9.5215602 | Down |
| NM_001256798 | NOL4L | 4.0027269 | 10.4774723 | Down |
| NM_022901 | LRRC19 | 2.567473 | 3.4883246 | Down |
| XR_243336 | LOC729652 | 2.3924719 | 3.6928647 | Down |
| NR_024100 | LINC00323 | 4.1571569 | 5.0330771 | Down |
| NM_002306 | LGALS3 | 3.7739806 | 3.4831365 | Down |
| NM_007015 | LECT1 | 2.2414925 | 3.0501088 | Down |
| NM_020802 | KIAA1377 | 3.1028204 | 11.7930583 | Down |
| NM_001282769 | KIAA1217 | 2.3047512 | 10.2743556 | Down |
| NM_001039792 | HRCT1 | 6.0386234 | 36.5943674 | Down |
| BC012091 | HES2 | 3.0238027 | 9.7456175 | Down |
| NM_021956 | GRIK2 | 4.3200284 | 3.6048787 | Down |
| NM_001005340 | GPNMB | 3.7370338 | 206.3936393 | Down |
| NM_004246 | GLP2R | 8.2462854 | 8.9927621 | Down |
| NM_000160 | GCGR | 2.01889 | 36.3501069 | Down |
| NM_004961 | GABRE | 2.9607312 | 84.743241 | Down |
| NM_199280 | FAM179A | 3.8170281 | 34.3988207 | Down |
| NM_001077710 | FAM110C | 2.7213439 | 15.0863166 | Down |
| NM_174912 | FAAH2 | 7.6530652 | 2.4905456 | Down |
| NM_004753 | DHRS3 | 2.0501894 | 42.3224429 | Down |
| NM_003389 | CORO2A | 2.2131558 | 10.4543539 | Down |
| NM_001850 | COL8A1 | 2.9891297 | 11.147959 | Down |
| NM_003465 | CHIT1 | 3.0397279 | 4.4913993 | Down |
| NM_207322 | C2CD4A | 5.3289286 | 15.7871265 | Down |
| NM_001195 | BFSP1 | 2.5234998 | 10.5263984 | Down |

**Supplementary Figure Legends**

**Supplementary Figure 1**

Quantitative real-time polymerase chain reaction (qRT-PCR) analysis of SMYD3 mRNA expression levels in 80 paired HCC and adjacent noncancerous tissue samples.

**Supplementary Figure 2**

Hep3B and SMMC-7721 cells were treated with cycloheximide (CHX, 20 μg/ml) for 0, 1 or 2 hours followed by western blotting for SMYD3.

**Supplementary Figure 3**

IGV (Integrative Genomics Viewer) profile of SMYD3-enriched regions over the S1PR1 locus which was mainly at the promoter regions (-1 kb to the TSS).

**Supplementary Figure 4**

IGV (Integrative Genomics Viewer) profile of SMYD3-enriched regions in the other 10 genes.

**Supplementary Figure 5**

Transwell assays were performed to evaluate cell migration in response to the knockdown of SMYD3 combined with the overexpression of S1PR1 in PLC/PRF/5 cells.
