# Supplementary figures and images for: SMYD3 promotes hepatocellular carcinoma progression by methylating S1PR1 promoters
Source: Cell Death Dis. 2021 Jul 23;12(8):731. doi: 10.1038/s41419-021-04009-8 (PMC8302584; doi:10.1038/s41419-021-04009-8)

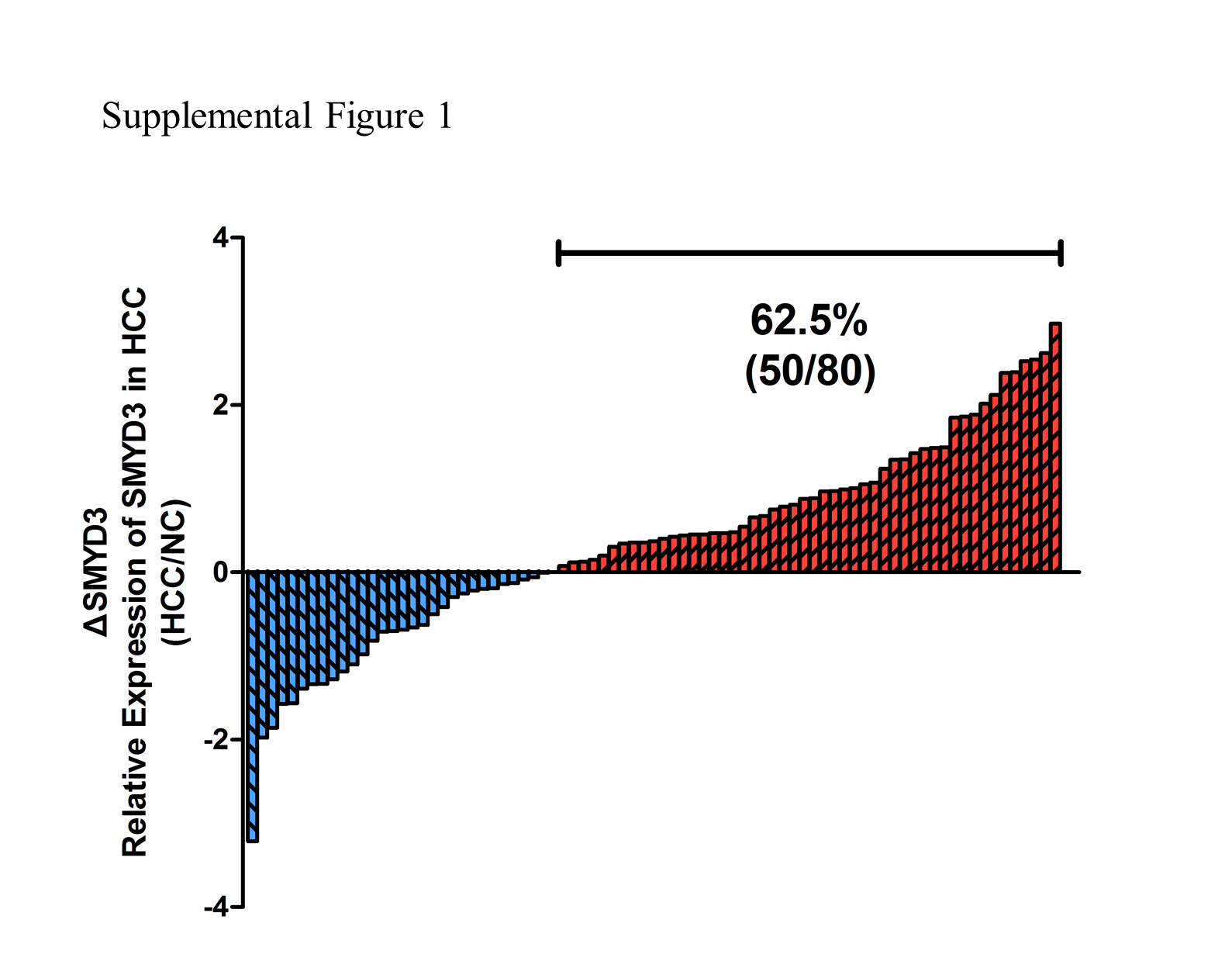

Supplement: Supplementary file 2 — Supplemental Figure 1 [file 41419_2021_4009_MOESM2_ESM.tif]

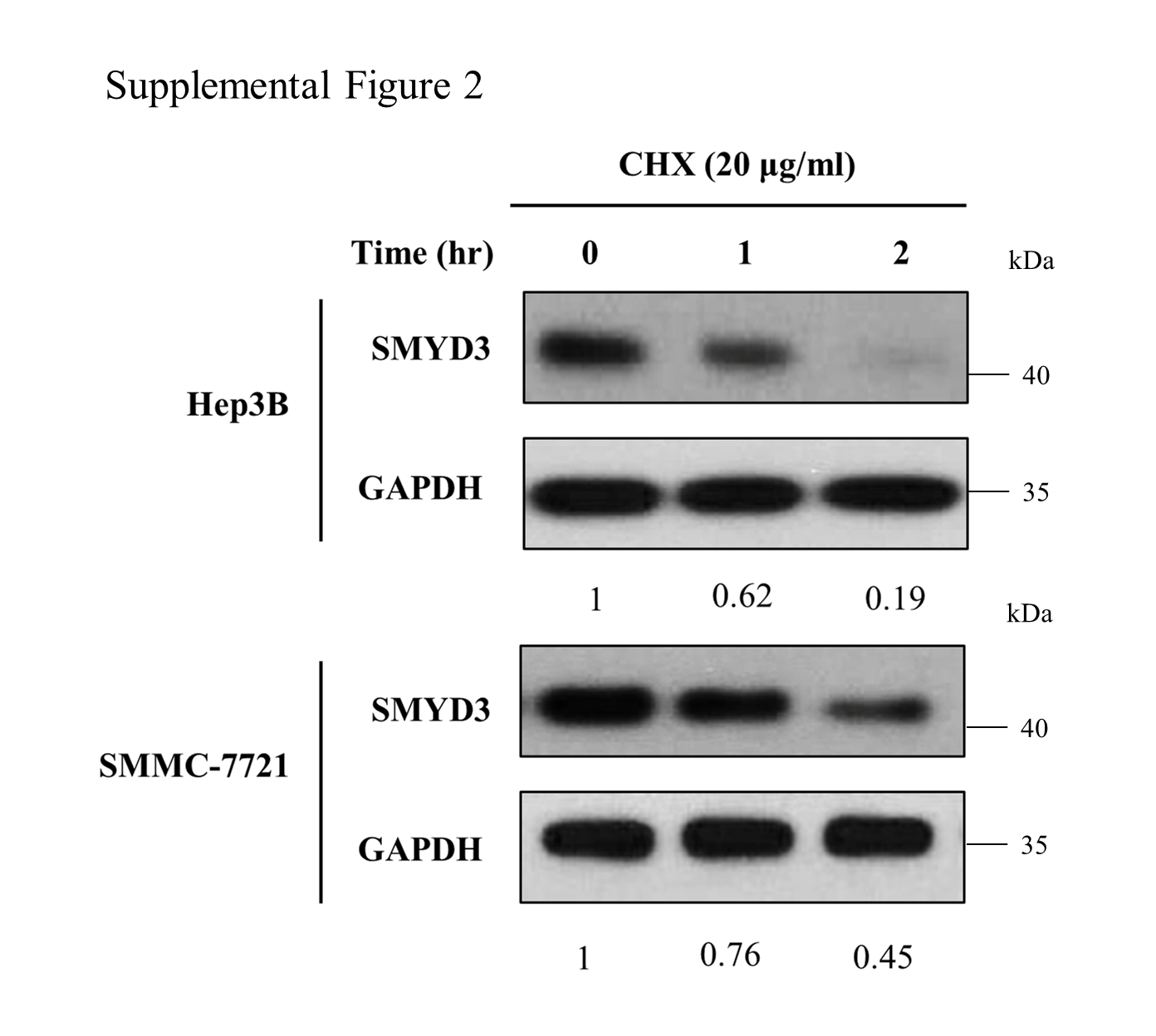

Supplement: Supplementary file 3 — Supplemental Figure 2 [file 41419_2021_4009_MOESM3_ESM.tif]

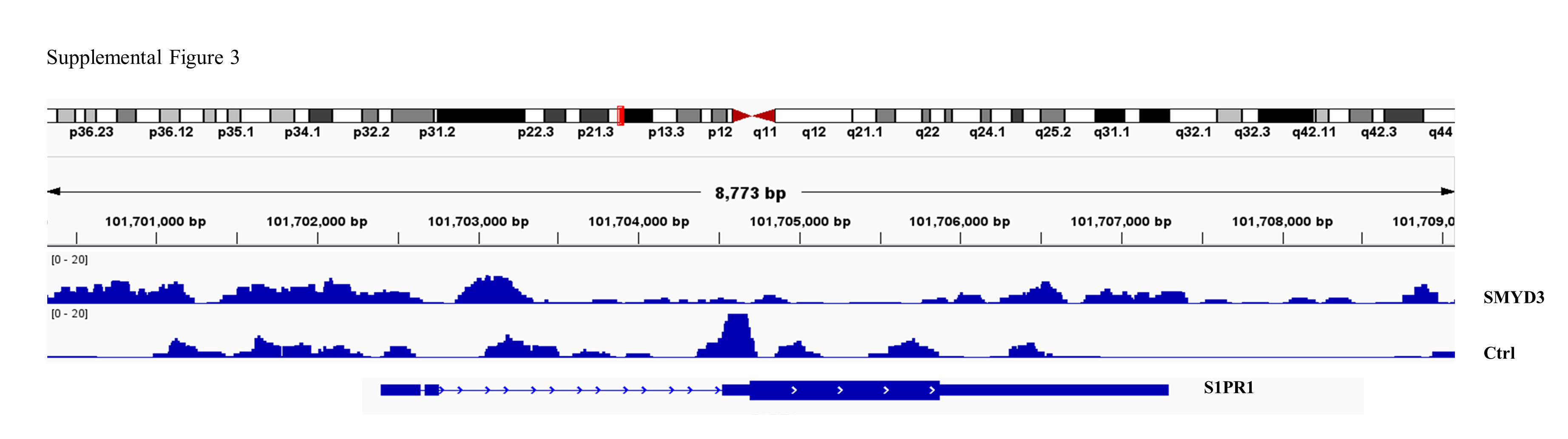

Supplement: Supplementary file 4 — Supplemental Figure 3 [file 41419_2021_4009_MOESM4_ESM.tif]

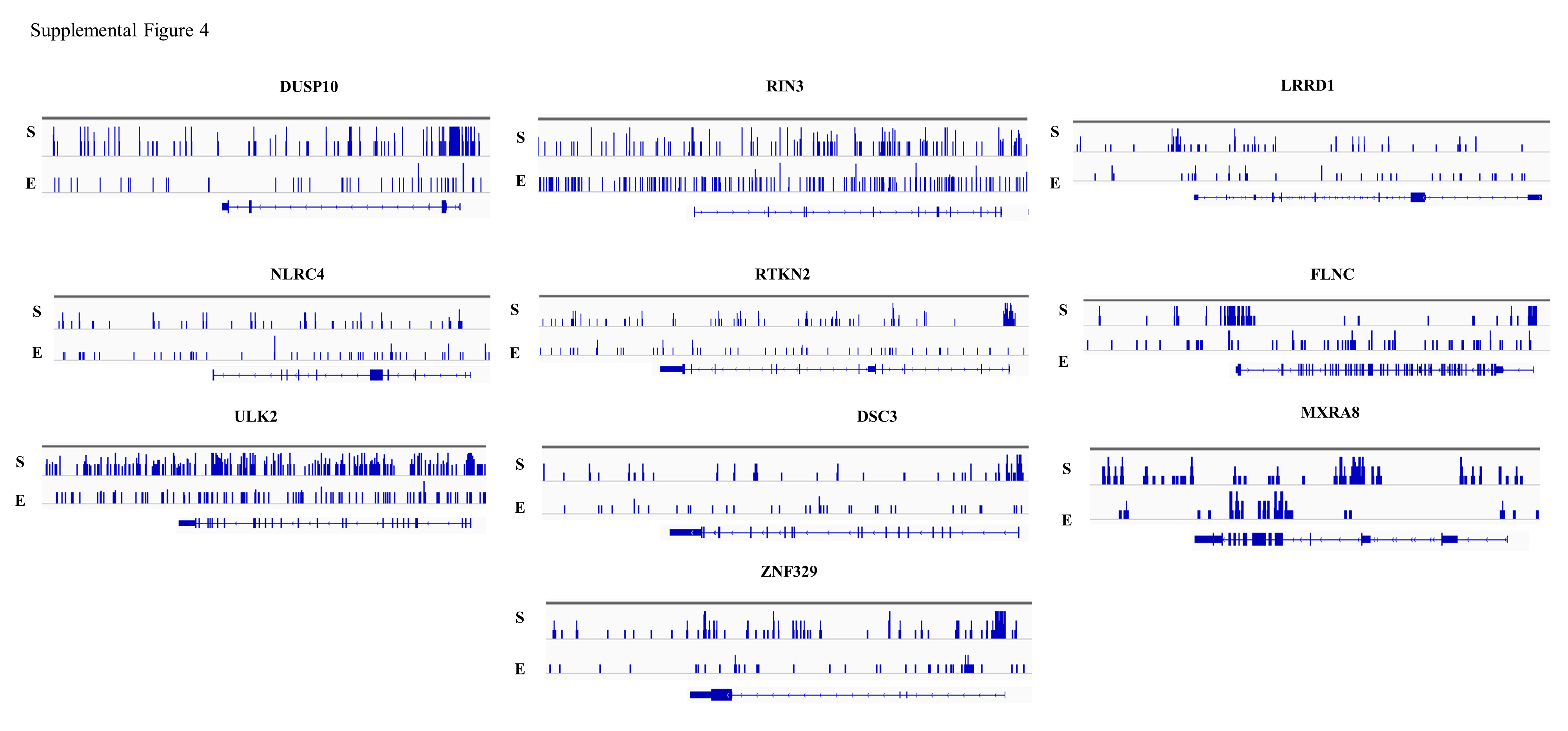

Supplement: Supplementary file 5 — Supplemental Figure 4 [file 41419_2021_4009_MOESM5_ESM.tif]

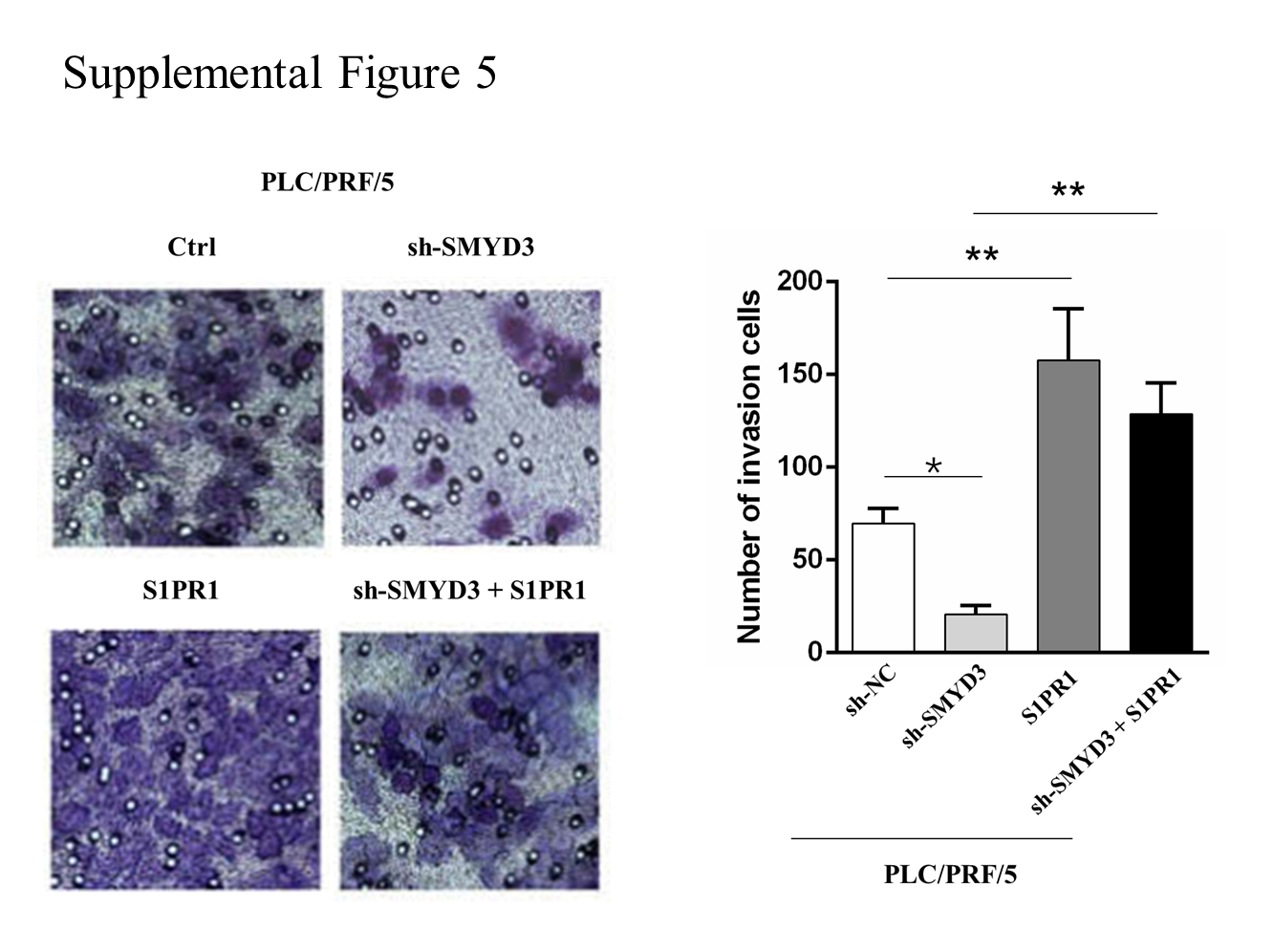

Supplement: Supplementary file 6 — Supplemental Figure 5 [file 41419_2021_4009_MOESM6_ESM.tif]
